# Endovascular management of internal carotid artery terminus aneurysms: a systematic review and meta-analysis

**DOI:** 10.1007/s10143-026-04210-3

**Published:** 2026-03-14

**Authors:** Mustafa Ismail, Rania H. Al-Taie, Ahmed AbdelWahab, Ahmad Abu Qdais, Hasna Loulida, Samip Patel, Norito Kinjo, Sami Al Kasab, Alejandro M. Spiotta

**Affiliations:** 1 Department of Neurosurgery, Division of Neuroendovascular Surgery, Medical University of South Carolina, Charleston, SC USA; 2 Department of Surgery, College of Medicine, Mustansiriyah University, Baghdad, Iraq; 3 Department of Neurology, Medical University of South Carolina, Charleston, SC USA

**Keywords:** Internal carotid artery, Terminus aneurysm, Endovascular treatment, Coil embolization, Stent-assisted coiling

## Abstract

Internal carotid artery (ICA) terminus aneurysms are a rare subtype of intracranial aneurysms with distinct hemodynamic characteristics and treatment challenges. This study aims to evaluate clinical outcomes associated with different endovascular approaches for ICA terminus aneurysms through a systematic review and meta-analysis. A comprehensive literature search through PubMed and Scopus from inception to July 2025. Data on patient demographics, aneurysm characteristics, and outcomes of coil embolization, stent-assisted coiling, and FD were extracted. Pooled analyses and subgroup comparisons were performed following PRISMA guidelines. Among 322 patients, primary coil embedding (59.0%) was the most used modality, followed by stent-assisted coiling (30.1%) and flow diversion (FD) (10.2%). A good functional outcome (mRS 0–2) was achieved in 74.5%, and complete occlusion in 72.4%. Primary coil embedding demonstrated significantly lower stroke (OR = 0.193, 95% CI: 0.056–0.662) and mortality (OR = 0.234, 95% CI: 0.045–0.906) compared to FD. Stent-assisted coiling achieved the highest rate of favorable outcomes (97.7%) and lowest recurrence (8.1%). Ruptured aneurysms were associated with worse outcomes, including lower occlusion (41–59%) and higher mortality (9.4%) than unruptured cases (occlusion: 94.5%, mortality: 2.0%). Endovascular strategies are commonly used in the management of ICA terminus aneurysms and demonstrate overall acceptable clinical and radiographic outcomes in contemporary practice. Observed differences among treatment modalities likely reflect underlying aneurysm morphology, clinical context, and treatment selection rather than intrinsic differences in device performance. Therefore, modality choice should be individualized based on anatomical and clinical considerations, and current findings should be interpreted as descriptive and hypothesis-generating.

**Supplementary Information:**

The online version contains supplementary material available at 10.1007/s10143-026-04210-3.

## Abstract (plain language summary)

**What is already known:**

ICA terminus aneurysms are uncommon (~2–9% of intracranial aneurysms), tend to present younger/smaller, and are driven by terminus hemodynamics; coiling is the historical mainstay, with FDs/intrasaccular options explored in selected anatomies, but comparative evidence is fragmented.

**What this study adds:**

This PRISMA-based review (Inception–Jul 2025) synthesizes 17 studies/322 patients and quantifies modality-specific outcomes: primary coil embedding (59%), stent-assisted coiling (30%), flow diversion (10%) with overall mRS 0–2 of 74.5% and complete occlusion 72.4%; primary coil embedding showed lower postoperative stroke (OR 0.193) and mortality (OR 0.234) versus flow diversion, while stent-assisted coiling achieved high good outcomes (97.7%) and low recurrence (8.1%).

**Impact:**

For ICA terminus aneurysms, endovascular therapy is widely used and associated with acceptable outcomes in contemporary practice. Apparent differences across treatment modalities likely reflect patient selection, aneurysm morphology, rupture status, and operator preference rather than intrinsic differences in treatment efficacy or safety. These findings support individualized treatment selection and highlight the need for prospective, rupture-stratified studies to better define optimal indications.

**Supplementary Information:**

The online version contains supplementary material available at 10.1007/s10143-026-04210-3.

## Introduction

Internal carotid artery (ICA) terminus aneurysms represent a distinct subtype of intracranial aneurysms, accounting for approximately 2%–9% of all cases [1, 2]. These aneurysms are particularly notable for occurring at a younger age and presenting at smaller sizes compared to aneurysms in other intracranial locations [3]. The wider terminus angles and intense hemodynamic stress in this region are believed to contribute to their early presentation and higher risk of rupture [4, 5]. Hemodynamic forces, particularly at arterial terminuss, play a crucial role in aneurysm development, growth, and rupture. Increased wall shear stress (WSS) near the aneurysm neck correlates with higher aspect ratio (AR) and size ratio (SR). These changes can lead to adverse flow [6].

Currently, endovascular coiling remains the primary treatment option for ICA terminus aneurysms. However, FDs and intrasaccular devices are being increasingly explored in selected cases, offering alternative strategies in anatomically complex or wide-neck aneurysms [7].

Despite recent advances in neurovascular technology, challenges persist in selecting the optimal treatment approach for ICA terminus aneurysms. Variations in aneurysm morphology, rupture status, and unique hemodynamic behavior at the ICA terminus often complicate decision-making. While prior studies have broadly addressed intracranial aneurysms, few have specifically focused on the ICA terminus aneurysms. Moreover, the evidence comparing outcomes across endovascular modalities remains fragmented. This review aims to address this gap by systematically analyzing the literature on ICA terminus aneurysms, with a particular focus on endovascular treatment strategies and clinical outcomes.

## Methods

### Study design and literature search strategy

This systematic review was conducted following the guidelines of the Preferred Reporting Items for Systematic Reviews and Meta-Analyses (PRISMA) statement [8]. A comprehensive search was conducted across two major biomedical databases, PubMed and Scopus, spanning the period from inception to July 2025. The search was restricted to English-language publications to ensure consistency and interpretability of clinical and methodological details.

The whole search string is: (stent-assisted coiling OR stent assisted coiling OR stent-assisted OR stent assisted OR flow diversion OR flow-diverting OR FD OR flow-diverter OR pipeline embolization device OR PED OR endovascular) AND (internal carotid artery terminus OR internal carotid terminus OR ICA terminus OR internal carotid artery terminus OR ICA terminus OR carotid terminus OR Carotid-T OR carotid T) AND (aneurysm OR aneurysms) in PubMed and (“stent assisted coiling” OR “stent assisted” OR “flow diversion” OR “FD” OR “pipeline embolization device” OR PED OR endovascular) AND (“internal carotid artery terminus” OR “internal carotid terminus” OR “ICA terminus” OR “internal carotid artery terminus” OR “ICA terminus” OR “carotid terminus” OR “carotid T”) AND (aneurysm OR aneurysms) in Scopus.

The literature search was conducted in PubMed and Scopus, which were selected for their comprehensive coverage. Additional databases such as Embase and the Cochrane Library were not searched due to resource and access limitations; however, reference lists of included studies were manually screened to minimize the risk of publication bias.

### Study screening and selection

All references identified from database searches were imported into Rayyan AI, a web-based platform for collaborative systematic review screening. Duplicates were automatically detected and manually verified. Two reviewers independently screened the titles and abstracts of the records. Discrepancies were resolved through discussion, and a third reviewer was consulted when consensus could not be reached.

### Eligibility criteria

Eligible studies included original research articles focusing on aneurysms located specifically at the ICA terminus. Both ruptured and unruptured aneurysms were considered. To maintain the clinical applicability of the findings, only studies that included data on endovascular treatment modality, clinical presentation, or outcomes were retained. Non-original articles such as review papers, editorials, expert commentaries, and conference abstracts were excluded. Case reports were only included if they offered unique clinical insights or described novel management strategies. Studies describing sidewall ICA aneurysms, paraclinoid lesions, or regional ICA aneurysms without clear involvement of the carotid bifurcation were excluded, even if identified during the initial search.

### Data extraction

Two authors independently extracted data using a predesigned standardized form. Extracted variables included study details (author, year, country, and design), sample size, patient demographics (mean age and gender), comorbidities, aneurysm presentation, rupture status, management approach (e.g., stent-assisted coiling, and flow diversion), and mean follow-up duration.

### Quality assessment

The methodological quality and risk of bias of included studies were assessed using validated tools. For observational cohort and case-control studies, the ROBINS-I (Risk of Bias In Non-randomized Studies - of Interventions) tool was used. It evaluates potential bias across seven domains, including confounding, participant selection, intervention classification, and outcome measurement [9]. For case reports and case series, quality was assessed using the CARE (Case Report) checklist, ensuring the appropriate reporting of patient history, clinical findings, intervention, and outcomes [10]. All assessments were conducted independently by two reviewers, with consensus reached through discussion in cases of disagreement.

### Statistical analysis

All quantitative analyses were conducted using Jamovi version 2.3, an open-source statistical platform based on the R statistical language. Descriptive statistics were used to summarize patient demographics, clinical characteristics, and treatment modalities.

For comparative meta-analyses of binary outcomes (e.g., complete occlusion, favorable clinical outcome), effect sizes were expressed as odds ratios (ORs) with corresponding 95% confidence intervals. Pooled estimates were calculated using a random-effects model. Between-study heterogeneity was assessed using the I² statistic and Cochran’s Q test. Forest plots were generated to visualize pooled effect estimates and heterogeneity.

## Results

### Study Characteristics

**Fig. 1:**
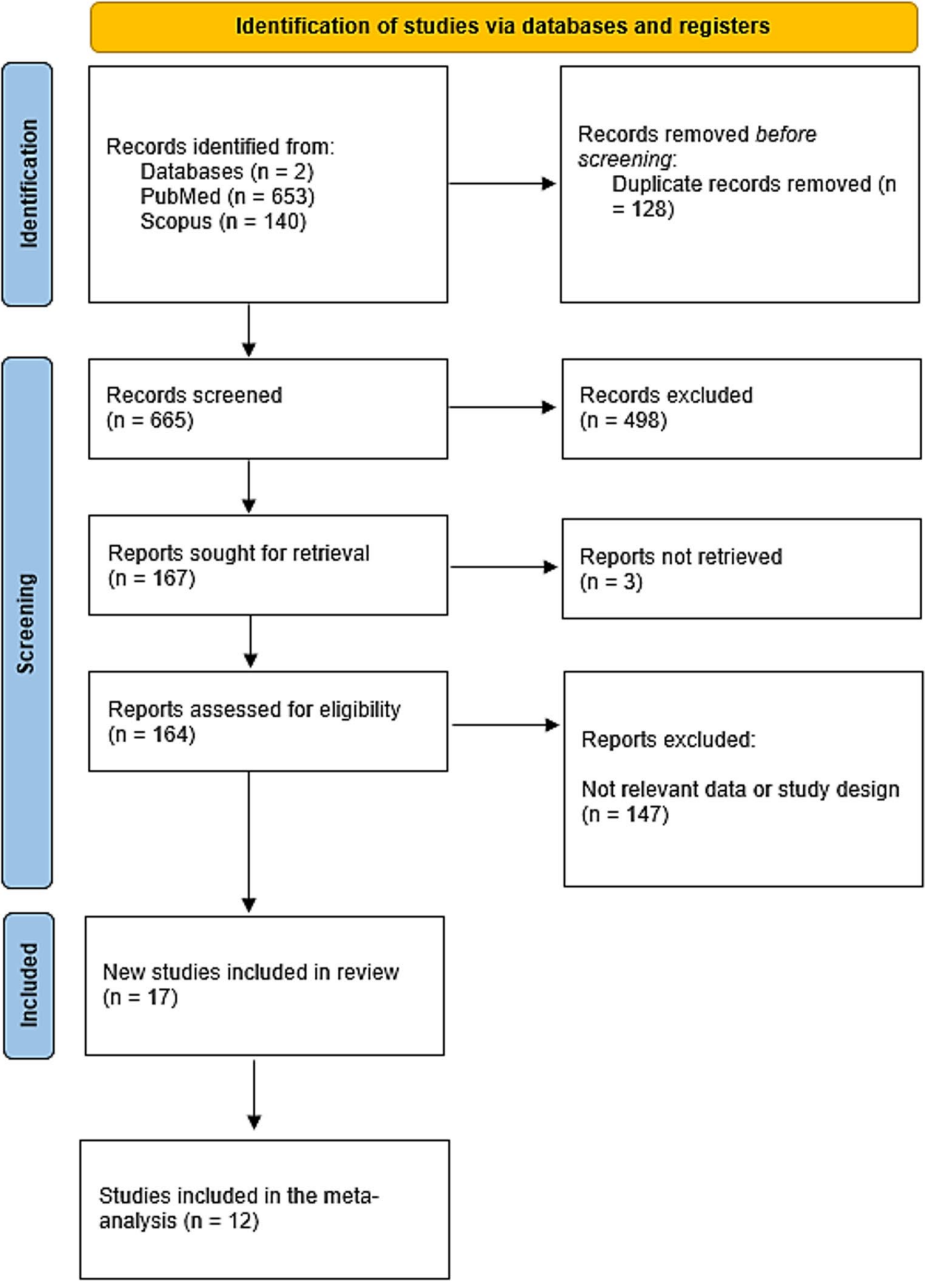
PRISMA Flowchart of the included studies

A total of 793 records were initially identified through database searches (PubMed, 653; Scopus, 140). After removing 128 duplicates, 665 records remained for screening, of which 498 were excluded based on titles and abstracts. Full-text retrieval was attempted for 167 reports, but 3 reports were inaccessible. This left 164 full-text articles evaluated for eligibility, and 147 were excluded due to irrelevant data or study design. Ultimately, 17 studies were included in the review, with 12 of those meeting the criteria for inclusion in the meta-analysis (Fig. 1).

The studies included a total of 322 patients diagnosed with ICA terminus aneurysms. The studies spanned across 12 countries, with the highest contributions from the USA, Japan, and India (Table 1) [11–25]. The mean age across studies ranged from 31.5 to 68 years, with most patients in their fifth to sixth decade of life. 124 (38.5%) were male and 194 (60.2%) were female, as reported in studies that specified sex. The remaining 4 patients had no reported gender data. Follow-up durations varied widely, from as short as 1 month in technical reports to over 50 months in some larger cohorts. However, long-term outcome data were limited across studies.

**Table 1:** Summary of Study Characteristics for Internal Carotid Terminus Aneurysms

Author(s)	Year	Country	Study Design	*N*	Mean Age (Years)	Males (*n*,%)	Co-morbidities	Presentation	Ruptured (*N*, %)	Management modality	Mean Follow-up (Months)
Benndorf et al. [11]	2005	USA	Technical Case Report	1	58	0 (0%)	Hypertension, hypothyroidism, hyperlipidemia	Mild headaches	0 (0%)	Stent-assisted coiling using cross-over technique	1
Sakamoto et al. [12]	2006	Japan	Retrospective cohort	8	63.0	4 (40%)	NR	SAH (5), asymptomatic (5)	5 (50%)	Coil embolization	NR
van Rooij et al. [13]	2008	Netherlands	Retrospective case series	46	49.3	13 (28%)	NR	SAH (26), incidental or mass effect (20)	26 (52%)	Coil embolization (50), 4 with balloon assistance	24.1
Uemura et al. [14]	2008	France	Retrospective case series	17	47.0	9 (53%)	NR	SAH (13), incidental (4)	13 (76%)	Coiling (17), 1 with balloon assistance	11.9
Oishi et al. [15]	2012	Japan	Retrospective case series	25	60.9	11 (44%)	NR	Incidental (23), SAH (2)	2 (8%)	Primary coiling (15), Balloon-assisted (9), Double catheter (1)	24.4
Zhang et al. [16]	2012	China	Case Report	1	39	1 (100%)	NR	Progressive chemosis	0 (0%)	Coil + Onyx embolization with balloon assist	3
Zhou et al. [17]	2012	China	Retrospective study	16	50.6	9 (56.25%)	NR	SAH (9), incidental (7)	9 (56.25%)	Stent-assisted coiling	12.1
Ban et al. [1]	2013	South Korea	Retrospective study	51	53.3	19 (37.3%)	Hypertension and multiple aneurysms were each present in 47.1% of patients, followed by dyslipidemia (27.5%), smoking (25.5%), and diabetes (11.8%).	SAH (12), incidental (32)	12 (23.5%)	Non-stent-assisted (*n* = 38), followed by single microcatheter (*n* = 23), stent-assisted (*n* = 13), multiple microcatheters (*n* = 10), and balloon-assisted (*n* = 5).	54.3
Lee et al. [18]	2014	South Korea	Retrospective study	65	54	23 (42.6%)	NR	SAH (12)	12 (22.2%)	Stent-assisted coil	27.3
Nossek et al. [19]	2014	USA	Retrospective case series	4	NR	NR	NR	Incidental	0 (0%)	Coiling (4); adjunctive stent-assisted coiling (1 case) and flow diversion (2 cases)	7
Morales-Valero et al. [2]	2014	USA	Retrospective study	36	50.7	14 (38.9%)	13 (36.1%) had additional aneurysms	9 SAH (25%), 3 symptomatic unruptured	9 (25%)	Endovascular coiling ± FD	40
Trivelato et al. [20]	2015	Brazil	Case report	1	64	0 (0%)	HTN, valve replacement	Visual loss, headache	0 (0%)	Pipeline device + coiling	6
Pira et al. [21]	2016	USA	Retrospective study	22	55.9	13 (59.1%)	Tobacco use (59.1%), hypertension (45.5%) and a positive family history (23.8%).	Headache, dizziness	1 (4.5%)	Primary coiling: *n* = 15 Balloon-assisted coiling: *n* = 1 FD-assisted coiling: *n* = 1	56.4
Mahajan et al. [22]	2018	India	Case report	1	66	0 (0%)	Hypertension	Embolic stroke	0 (0%)	Stent-assisted coiling	NR
Cagnazzo et al. [23]	2020	France	Retrospective study	20	53	4 (20%)	NR	Mass effect, cranial nerve palsy	6 (30%)	Flow-diverter ± coils	14.3
Mahmoud et al. [24]	2020	Egypt	Case series	7	48.7	3 (42.9%)	NR	Headache: 3 patients (42.9%) SAH: 2 patients (28.6%)	2 (28.6%)	Flow-diverter	6
Spinelli et al. [25]	2022	USA	Case report	1	78	1 (100%)	NR	Blurred vision	0 (0%)	Endovascular coiling + flow diverting stent	2

NR indicates outcomes that were not reported in the original studies. These variables were therefore unavailable for extraction and were not included in the corresponding pooled analyses

The most common comorbidity was hypertension, reported in 42 patients (13.0%). Dyslipidemia was the next most frequent in 15 patients (4.7%), followed by diabetes mellitus (DM) in 6 patients (1.9%). A family history of aneurysms was noted in 5 patients (1.6%). No patients had documented connective tissue disorders or vasculopathy (0%). Initial clinical grading was variably reported. The Glasgow Coma Scale (GCS) was documented in four patients, all scores of 15. Hunt and Hess grading was reported in seven studies, with most scores being grades I and II. One study reported 17 patients with grades I–II, 6 with grade III, and 3 with grades IV–V. Another study noted distributions across all five grades. The World Federation of Neurological Societies (WFNS) grade was not reported. No pediatric cases were identified.

### Quality assessment results

The quality assessment using the CARE and ROBINS-I tools revealed that most case reports and observational studies had moderate risk of bias, with a few showing serious concerns mainly due to confounding and selective reporting. Further details are summarized in Tables S1 and S2.

### Aneurysm characteristics and clinical presentation

Among the 322 ICA terminus aneurysms, laterality was nearly evenly split: 122 (37.9%) on the right, 123 (38.2%) on the left, and one case (0.3%) bilateral. Laterality was unspecified in 76 cases (23.6%) due to lack of reporting. Aneurysm sizes were variably reported; one case had a recorded size of 0 mm, likely a typo or thrombosed. Thrombus was reported in 2 patients (0.6%). Intracerebral hemorrhage (ICH) was the initial feature in 91 patients (28.3%), with no documented ischemic strokes.

### Symptomatology at presentation

Headache was the most common presenting symptom, occurring in 86 individuals (26.7%). Visual disturbances were reported in 4 patients (1.2%). Notably, there were no documented cases of decreased level of consciousness (DLOC), cranial nerve palsy, paralysis, or seizures in the included studies.

### Treatment outcomes

Of the 322 ICA terminus aneurysm cases included, primary coil embedding embolization was most common, performed in 190 patients (59.0%). Stent-assisted coiling was used in 97 cases (30.1%), and FD, alone or with coiling, in 33 patients (10.2%). Clinical outcomes were reported for most patients. A good outcome (mRS 0–2) was achieved in 240 patients (74.5%), and complete aneurysm occlusion was documented in 233 cases (72.4%). Rebleeding occurred in 2 patients (0.6%). Aneurysm recurrence was observed in 42 patients (13.0%), and Postoperative ischemic stroke in 19 cases (5.9%). Overall mortality was 2.8%, with 9 deaths.

### Proportional analysis of treatment outcomes in stent-assisted cohorts

**Fig. 2:**
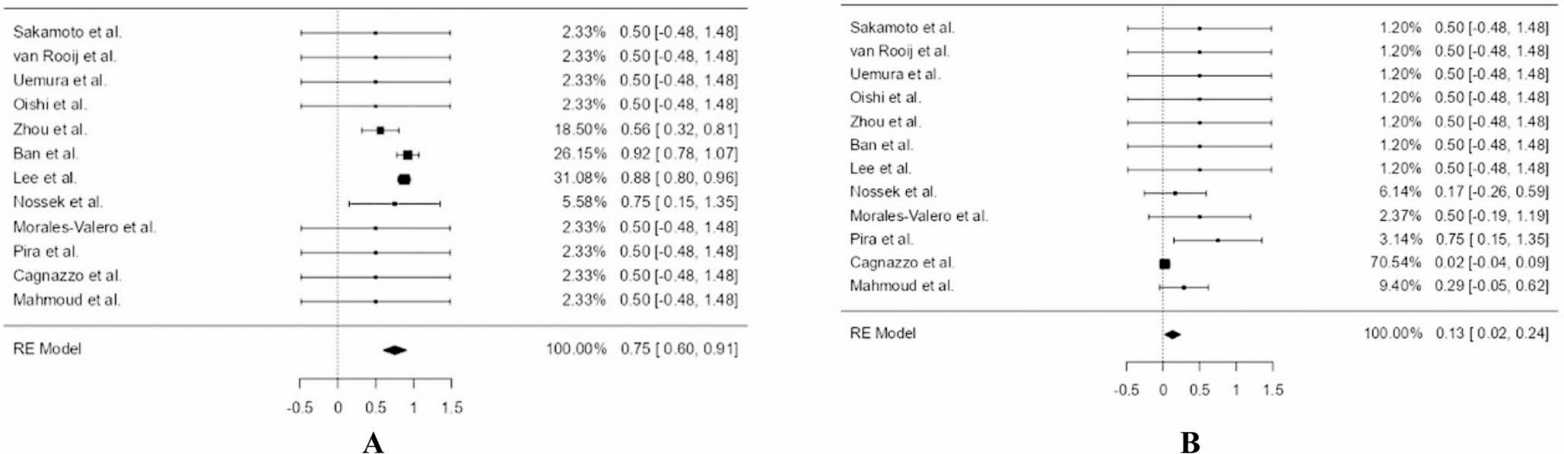
**(A)** Forest plot showing the proportion of complete aneurysm occlusion in patients treated with stent-assisted coiling for ICA terminus aneurysms. The pooled estimate using a random-effects model was 75% (95% CI: 0.59–0.91), with moderate heterogeneity across studies. **(B)** Forest plot illustrating the proportion of Postoperative ischemic stroke in patients treated with FDs for internal carotid artery terminus aneurysms. The pooled estimate using a random-effects model was 13% (95% CI: 0.02–0.24), with low to moderate heterogeneity across studies

Pooled estimates from 12 studies evaluating stent-assisted endovascular treatment in ICA terminus aneurysms revealed a recurrence rate of 8.1% (95% CI: 0.031–0.132, *p* = 0.002), with no observed heterogeneity across studies (I² = 0%). A good functional outcome (modified Rankin Scale 0–2) was achieved in 97.7% of patients treated with stent-assisted approaches (95% CI: 0.950–1.005, *p* < 0.001), again with no heterogeneity (I² = 0%). The complete aneurysm occlusion rate was 75.3% (95% CI: 0.598–0.909, *p* < 0.001), with moderate heterogeneity across studies (I² = 42.8%) (Fig. 2A). Postoperative ischemic stroke occurred in 7.6% of patients (95% CI: 0.027–0.125, *p* = 0.002), while the pooled mortality rate was 2.2% (95% CI: − 0.005 to 0.049, *p* = 0.110). Importantly, no cases of rebleeding were reported in the stent-treated population.

### Proportional analysis of treatment outcomes in FD cohorts

In the FD subgroup, pooled analysis of 12 studies demonstrated a recurrence rate of 11.9% (95% CI: − 0.004–0.242, *p* = 0.058), with mild heterogeneity (I² = 18.7%). A good functional outcome (modified Rankin Scale 0–2) was achieved in 78.6% of patients (95% CI: 0.619–0.953, *p* < 0.001), with moderate heterogeneity observed (I² = 32.2%). The complete aneurysm occlusion rate was 74.0% (95% CI: 0.558–0.923, *p* < 0.001), and Postoperative ischemic stroke occurred in 13.1% of cases (95% CI: 0.023–0.239, *p* = 0.017) (Fig. 2B). The mortality rate was 18.1% (95% CI: 0.025–0.336, *p* = 0.023), and rebleeding was reported in 18.1% of patients (95% CI: 0.025–0.336, *p* = 0.023). Heterogeneity was moderate across these outcomes (I² ranging from 32% to 38%).

### Proportional analysis of treatment outcomes in primary coil embedding cohorts

Among patients treated with primary coil embedding, pooled estimates from 12 studies showed a recurrence rate of 13.6% (95% CI: 0.040–0.232, *p* = 0.006), with substantial heterogeneity (I² = 84.4%). A good functional outcome was achieved in 74.2% of patients (95% CI: 0.544–0.940, *p* < 0.001), though heterogeneity was high (I² = 95.3%). The complete aneurysm occlusion rate was 72.3% (95% CI: 0.557–0.889, *p* < 0.001), also with high heterogeneity (I² = 89.0%). Postoperative ischemic stroke occurred in 4.3% of cases (95% CI: 0.015–0.071, *p* = 0.003), and the mortality rate was 2.7% (95% CI: 0.004–0.049, *p* = 0.019). Rebleeding was rare, reported in only 1.8% of patients (95% CI: 0.000–0.036, *p* = 0.053), with no heterogeneity across studies (I² = 0%).

### Comparative Outcomes: FD vs. Stent-Assisted Coiling

A direct comparison between FD and stent-assisted strategies revealed no statistically significant differences in key outcomes. The odds of achieving a good functional outcome (mRS 0–2) were lower in the FD group compared to stents, but the difference was not significant (OR = 0.693, 95% CI: 0.171–2.804, *p* = 0.607). Similarly, complete aneurysm occlusion showed no significant advantage for either modality (OR = 0.869, 95% CI: 0.229–3.292, *p* = 0.836). Postoperative ischemic stroke was more frequent in the FD group, though again not statistically different (OR = 1.506, 95% CI: 0.381–5.951, *p* = 0.559). The mortality rate also trended higher with FD (OR = 1.424, 95% CI: 0.322–6.295, *p* = 0.641), although this difference did not reach statistical significance.

### Comparative outcomes: primary coil embedding vs. stent-assisted coiling

A good functional outcome, defined as a modified Rankin Scale (mRS) score of 0–2, was observed more frequently in the primary coil embedding group (OR = 1.188, 95% CI: 0.364–3.881, *p* = 0.776), although the difference was not statistically significant. Similarly, the rate of complete aneurysm occlusion was higher among primary coil embedding patients (OR = 1.502, 95% CI: 0.478–4.720, *p* = 0.487), but this difference also failed to reach statistical significance. Postoperative ischemic stroke occurred less frequently in the coiling cohort, with an odds ratio of 0.341 (95% CI: 0.101–1.152, *p* = 0.083), however, this did not achieve statistical significance. Mortality rates were likewise lower in the primary coil embedding group compared to stent-assisted coiling, with an odds ratio of 0.336 (95% CI: 0.081–1.400, *p* = 0.134), but again without statistical significance. Heterogeneity across all four outcomes was minimal (I² = 0% for functional outcome, occlusion, and stroke; I² = 16.2% for mortality).

### Comparative outcomes: primary coil embedding vs. FD

**Fig. 3:**
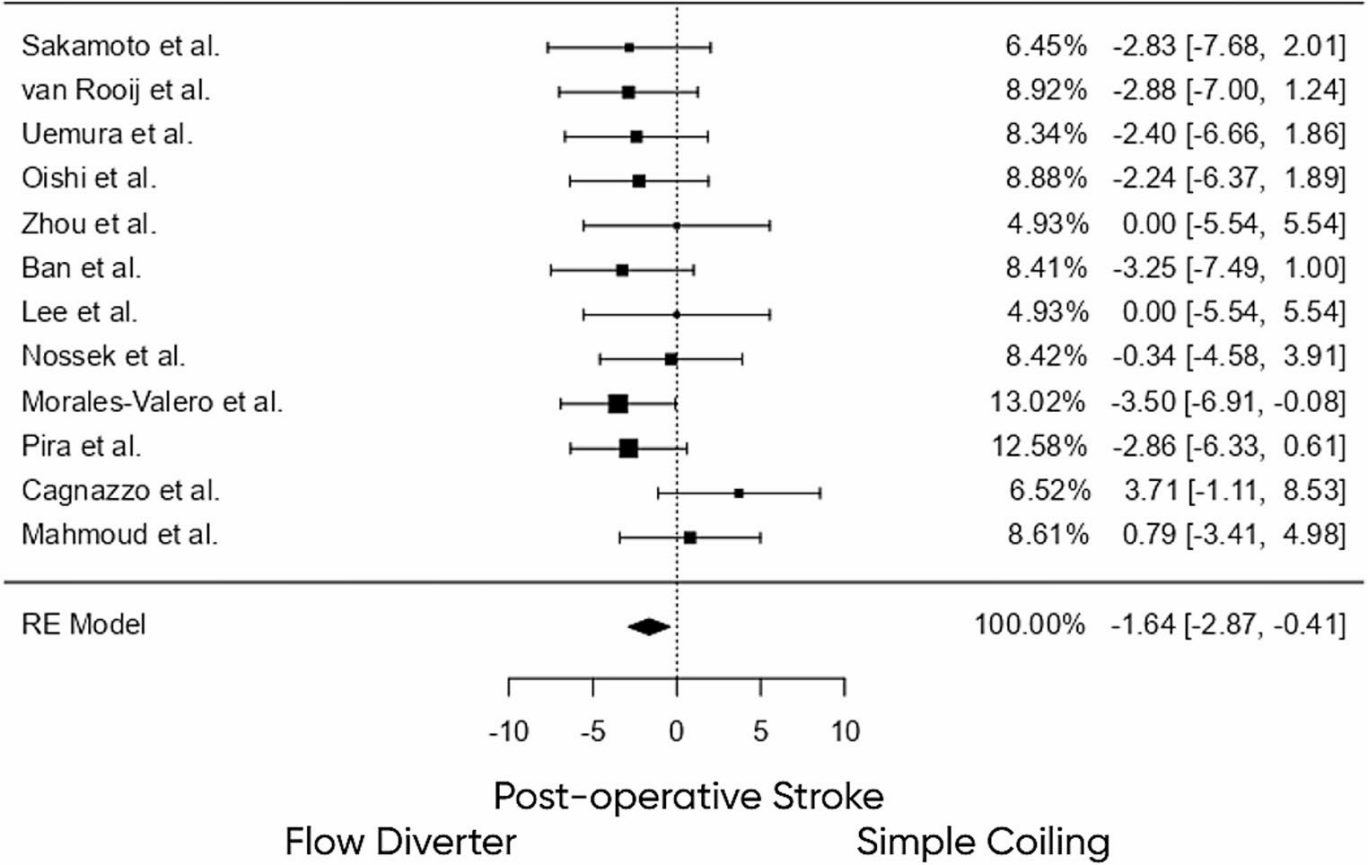
Forest plot comparing Postoperative ischemic stroke rates between patients treated with FDs and those treated with coil embolization for internal carotid artery terminus aneurysms. Negative values indicate a lower risk of Postoperative ischemic stroke in the coiling group. The pooled effect estimate using a random-effects model demonstrated a significant reduction in Postoperative ischemic stroke with coiling compared to FD (OR: − 1.64; 95% CI: − 2.87 to − 0.41), odds ratio of 0.193 (95% CI: 0.056–0.662, *p* = 0.009)

A good functional outcome (mRS 0–2) was more frequently achieved in the primary coil embedding group (OR = 1.541, 95% CI: 0.404–5.882, *p* = 0.527), though this difference did not reach statistical significance. Similarly, complete aneurysm occlusion was more likely with primary coil embedding (OR = 1.501, 95% CI: 0.466–4.835, *p* = 0.496), but again the difference was not significant. Importantly, Postoperative ischemic stroke occurred significantly less often in the Primary coil embedding cohort, with an odds ratio of 0.193 (95% CI: 0.056–0.662, *p* = 0.009), indicating a statistically meaningful reduction in ischemic complications compared to FD (Fig. 3**)**. Mortality was also significantly lower in patients treated with coiling (OR = 0.234, 95% CI: 0.045–0.906, *p* = 0.036). Heterogeneity across outcomes was generally low (I² = 0%–15%).

### Comparison between ruptured and unruptured ICA terminus aneurysms

In the rupture-only subgroup (k = 12 studies), the pooled recurrence rate was 18.6% (95% CI: 0.090–0.282; I² = 34.9%). Rebleeding occurred in 4.9% (95% CI: 0.007–0.091; I² = 0%). A good functional outcome (mRS 0–2) was achieved in 84.9% (95% CI: 0.770–0.928; I² = 16.9%). Complete occlusion was observed in 58.9% (95% CI: 0.396–0.783), with high heterogeneity (I² = 82.0%). We conducted a sensitivity analysis. After excluding outlier studies, the pooled complete occlusion rate was 41.4% (95% CI: 0.221–0.607), with substantially reduced heterogeneity (I² = 48.6%, *p* = 0.062). Post-operative stroke occurred in 5.1% (95% CI: 0.008–0.093; I² = 0%), and mortality was 9.4% (95% CI: 0.037–0.150; I² = 0%).

In the unruptured subgroup (k = 12 studies), the pooled recurrence rate was 13.5% (95% CI: 0.042–0.229; I² = 89.1%). Rebleeding was rare, occurring in 1.6% (95% CI: 0.001–0.032; I² = 0%). A good functional outcome (mRS 0–2) was achieved in 97.3% (95% CI: 0.951–0.994; I² = 5.9%). The pooled complete occlusion rate before sensitivity analysis was 72.0% (95% CI: 0.565–0.875), although heterogeneity was very high (I² = 93.9%). To address this, a sensitivity analysis was conducted, which yielded a revised pooled complete occlusion rate of 94.5% (95% CI: 0.905–0.985) with no observed heterogeneity (I² = 0%, *p* = 0.961). Post-operative stroke occurred in 2.0% (95% CI: 0.002–0.037; I² = 0%), while mortality was also 2.0% (95% CI: 0.003–0.038; I² = 0%).

When directly comparing outcomes between ruptured and unruptured aneurysms, recurrence was not significantly different (OR 2.48, 95% CI: 0.80–7.66; *p* = 0.116). However, the risk of rebleeding was significantly higher in ruptured cases (OR 3.45, 95% CI: 1.10–10.83; *p* = 0.034). Good functional outcome was less likely following rupture (OR 0.22, 95% CI: 0.09–0.53; *p* < 0.001), while complete occlusion rates did not differ significantly (OR 0.60, 95% CI: 0.29–1.24; *p* = 0.167). Mortality was significantly higher among ruptured aneurysms (OR 3.74, 95% CI: 1.43–9.77; *p* = 0.007).

### Subgroup meta-analysis of outcomes in ruptured and unruptured ICA terminus aneurysms

In the pooled proportional meta-analysis, unruptured ICA terminus aneurysms treated with simple coiling showed a recurrence rate of 15.9% (95% CI: 0.066–0.252; I² = 70.3%) and complete occlusion in 79.0% (95% CI: 0.664–0.915). Good functional outcome was achieved in 96.8% (95% CI: 0.944–0.993), while Postoperative ischemic stroke and mortality were low at 2.1% and 1.7%, respectively. For unruptured aneurysms treated with stent-assisted coiling, recurrence was lower (6.8%, 95% CI: − 0.013–0.150) with a complete occlusion rate of 71.1% (95% CI: 0.559–0.862) and good outcome in 89.0% (95% CI: 0.787–0.994). In ruptured cases, recurrence was higher, reaching 16.5% with simple coiling and 36.4% with stent-assisted coiling. Good functional outcome ranged from 82.7% with simple coiling to 86.7% with stent-assisted coiling. Mortality was 10.0% in the simple coiling group and 13.3% in the stent-assisted group (Table 2).

**Table 2:** Pooled Proportional Meta-Analysis of Clinical and Radiological Outcomes in Ruptured and Unruptured ICA Terminus Aneurysms Treated with Simple Coiling or Stent-Assisted Coiling

Category	Outcome	Proportional Rate	95% CI	*p*-value	I² (%)
**Ruptured ICA Terminus Aneurysms Treated with Simple Coiling**	Recurrence	16.5%	0.065–0.265	0.001	34.5
	Rebleeding	6.5%	0.015–0.116	0.012	0
	Good functional outcome (mRS 0–2)	82.7%	0.747–0.907	< 0.001	0
	Complete occlusion	66.1%	0.433–0.888	< 0.001	87.4
	Postoperative ischemic stroke	5.2%	0.006–0.097	0.027	0
	Mortality	10.0%	0.038–0.162	0.002	0
**Ruptured ICA Terminus Aneurysms Treated with Stent-Assisted Coiling**	Recurrence	36.4%	0.143–0.585	0.001	0
	Rebleeding	13.3%	−0.003–0.269	0.056	0
	Good functional outcome (mRS 0–2)	86.7%	0.731–1.003	< 0.001	0
	Complete occlusion	86.7%	0.731–1.003	< 0.001	0
	Postoperative ischemic stroke	13.3%	−0.003–0.269	0.056	0
	Mortality	13.3%	−0.003–0.269	0.056	0
**Unruptured ICA Terminus Aneurysms Treated with Simple Coiling**	Recurrence	15.9%	0.066–0.252	< 0.001	70.3
	Rebleeding	1.6%	−0.001–0.034	0.065	0
	Good functional outcome (mRS 0–2)	96.8%	0.944–0.993	< 0.001	0
	Complete occlusion	79.0%	0.664–0.915	< 0.001	86.3
	Postoperative ischemic stroke	2.1%	0.001–0.042	0.038	1.4
	Mortality	1.7%	−0.001–0.035	0.059	0
**Unruptured ICA Terminus Aneurysms Treated with Stent-Assisted Coiling**	Recurrence	6.8%	−0.013–0.150	0.0996	0.0
	Rebleeding	6.8%	−0.013–0.150	0.0996	0.0
	Good functional outcome (mRS 0–2)	89%	0.787–0.994	< 0.0001	0.0
	Complete occlusion	71%	0.559–0.862	< 0.0001	0.0
	Postoperative ischemic stroke	11%	0.006–0.213	0.0380	0.0
	Mortality	6.8%	−0.013–0.150	0.0996	0.0

Confidence intervals for pooled proportions were derived from random-effects models estimated on a transformed scale and subsequently back-transformed to the proportion scale. In subgroups with very low event rates and small sample sizes, this approach may yield lower confidence bounds that extend slightly below zero. These values reflect statistical uncertainty after back-transformation and do not indicate biologically meaningful negative risks. No normal-approximation (Wald) method was used

When comparing ruptured ICA terminus aneurysms treated with stent-assisted coiling versus simple coiling, no significant differences were observed in good functional outcome (OR 0.44, 95% CI: 0.11–1.82; *p* = 0.255) or complete occlusion (OR 0.68, 95% CI: 0.16–2.93; *p* = 0.610). Mortality showed a trend toward higher odds with stent-assisted coiling (OR 3.29, 95% CI: 0.76–14.32; *p* = 0.113), although this did not reach statistical significance. Heterogeneity across all comparisons was low (I² = 0%). In the subgroup of unruptured ICA terminus aneurysms, stent-assisted coiling was associated with significantly lower odds of achieving good functional outcome compared with simple coiling (OR 0.18, 95% CI: 0.05–0.67; *p* = 0.010). No significant difference was observed in complete occlusion rates (OR 0.66, 95% CI: 0.22–1.94; *p* = 0.446). However, stent-assisted coiling was associated with a significantly higher mortality risk (OR 6.68, 95% CI: 1.61–27.61; *p* = 0.009). Heterogeneity across all outcomes was minimal (I² = 0%).

## Discussion

In the endovascular management of carotid terminus aneurysms, coil embolization remains the most commonly reported treatment approach in the literature, with generally acceptable safety outcomes. However, observed differences among treatment modalities should be interpreted in the context of aneurysm characteristics, rupture status, and treatment selection rather than as evidence of comparative superiority. Importantly, the unique hemodynamic environment of the ICA terminus, characterized by bifurcation geometry with dual outflow into the anterior and middle cerebral arteries, persistent inflow jets, and competitive flow patterns, plays a central role in treatment durability and complication profiles. These flow dynamics may contribute to coil compaction and recurrence and can limit the effectiveness of FD at bifurcation sites, while stent placement across the terminus may alter ACA–MCA flow balance in unpredictable ways with potential ischemic implications. Together, these anatomical and physiological considerations provide a mechanistic framework for interpreting the heterogeneous outcome patterns observed in this analysis [23, 26, 27].

Overall, endovascular treatment had favorable results, with 74.5% of patients achieving a good functional outcome (mRS 0–2) and 72.4% complete aneurysm occlusion. Stent-assisted coiling showed the best clinical outcomes (97.7%) and lowest recurrence (8.1%). Primary coil embedding had significantly lower stroke and death rates than FD. No modality was clearly superior across all metrics; coil embolization offers the best safety, with stent-assisted techniques being effective in selected cases. The high favorable outcome rate with stent-assisted coiling may reflect selection bias, as it’s often used for unruptured aneurysms with favorable anatomy and stable status, which skews results. Patients with ruptured aneurysms or comorbidities are underrepresented due to antiplatelet concerns. Many studies had small samples, non-randomized, retrospective designs, limiting generalizability, and publication bias may favor positive outcomes. Thus, while efficacy appears high, these factors must be considered in real-world contexts. Higher complication rates with FDs likely stem from treating complex, wide-necked, or fusiform aneurysms, which carry more procedural risk [28]. Some studies included ruptured aneurysms where DAPT increases hemorrhage risk. Cohorts often consisted of older patients, heightening thromboembolic risk. These factors, along with small sample sizes and selection bias, probably explain higher complications. Future studies should stratify outcomes based on rupture status, age, and antiplatelet use risks.

The review highlights that hypertension was the most prevalent comorbidity among patients with ICA terminus aneurysms, followed by dyslipidemia and diabetes mellitus. Notably, no cases of connective tissue disorders or vasculopathies were reported, and a positive family history of aneurysms was rare. Initial clinical grading was inconsistently documented across studies; however, the majority of patients presented in good clinical condition. For instance, Morales-Valero et al. [2] reported that the patient had a GCS score of 15, while Ban et al. [1] observed that most patients had Hunt and Hess grades I–II. No pediatric cases were identified in any of the included studies.

Primary coil embedding embolization was the most common endovascular method, used in nearly 60% of cases. Despite challenges at the ICA terminus, stent-assisted coiling and FD were effective in selected patients. Overall, outcomes were favorable, with 74.5% achieving good functional results (mRS 0–2) and 72.4% complete occlusion. Severe complications were rare: rebleeding 0.6%, stroke 5.9%, mortality 2.8%. These findings support the safety and effectiveness of endovascular treatments, though choices should be tailored to aneurysm and patient factors. Stent-assisted coiling had the best safety and efficacy, with 97.7% good outcomes, no rebleeding, 8.1% recurrence, and 2.2% mortality. The mortality observed in the FD subgroup should be interpreted with caution. FD cases in the included studies frequently involved ruptured aneurysms, complex or previously treated lesions, early-generation devices, and heterogeneous antiplatelet regimens, all of which may have contributed to the observed outcomes. These factors limit the generalizability of the reported mortality signal and do not reflect contemporary elective FD practice at the ICA terminus. Accordingly, the present findings are descriptive and hypothesis-generating and should not be extrapolated as indicative of intrinsic risk associated with modern FD.

The distinction between ruptured and unruptured aneurysms is critical as they are different entities with different risks. Our primary analyses pooled outcomes from both, but this may hide nuances. Ruptured aneurysms are treated urgently with higher risks like vasospasm and rebleeding, while unruptured aneurysms are managed electively with better neurological status [29]. Future studies should stratify by rupture status, though current data limit this. Most favorable outcomes were in unruptured cases, partly explaining their high efficacy and low complications. Direct comparisons of endovascular strategies showed no statistically significant differences in outcomes like functional status, aneurysm occlusion, stroke, or death; trends favored stent-assisted coiling. Primary coil embedding had higher odds of good outcomes and aneurysm occlusion but was not significant, though it showed a trend toward lower stroke and mortality. Compared to FDs, coiling significantly reduced Postoperative ischemic stroke (OR = 0.193, *p* = 0.009) and death (OR = 0.234, *p* = 0.036), suggesting fewer ischemic issues and better survival.

Our subgroup analysis highlights rupture status as a pivotal determinant of treatment outcomes in ICA terminus aneurysms. Patients with unruptured aneurysms exhibited significantly better clinical and angiographic results, including higher rates of good functional outcome (97.3%), complete occlusion (94.5%), and lower risks of stroke and mortality (2.0%), in contrast to their ruptured counterparts, who experienced reduced occlusion rates (41–59%) and markedly higher mortality (9.4%). These outcome disparities are supported by hemodynamic modeling studies that suggest biomechanical and flow-related factors may exacerbate the risks in ruptured cases. Rostamian et al. demonstrated that stent-induced deformation of aneurysm morphology significantly reduces intra-aneurysmal wall shear stress (WSS) by up to 71%, compared to only 20% reduction with coiling alone, implying that morphological stabilization plays a critical role in rupture prevention [30]. This may partially explain why endovascular treatments appear more efficacious in unruptured lesions, where the aneurysmal wall remains biomechanically intact and more responsive to flow-altering interventions. In line with this, Abbasi et al. [31] reported that ruptured aneurysms often present with more complex geometries and higher inflow rates, increasing the difficulty of achieving complete occlusion and durable outcomes through coiling alone. Moreover, ruptured aneurysms may already exhibit compromised vascular integrity, limiting the effectiveness of endovascular techniques optimized for more stable lesions.

Antiplatelet therapy represents a critical and variably reported factor influencing outcomes in stent-assisted coiling and FD [32, 33]. Across the included studies, antiplatelet regimens, duration of dual antiplatelet therapy, and the use of platelet function testing were heterogeneous and often incompletely described. Such variability may contribute to differences in ischemic and hemorrhagic complications, particularly in ruptured cases where antiplatelet tolerance is limited. These factors are unlikely to be fully captured in retrospective series and may partially account for observed stroke and mortality rates. Accordingly, antiplatelet-related considerations should be recognized as an important limitation when interpreting outcomes of stent-based endovascular strategies at the ICA terminus.

Intrasaccular flow-disruption devices have emerged as an important contemporary option for wide-neck bifurcation aneurysms, including selected ICA terminus lesions, offering treatment without permanent intraluminal implants or prolonged dual antiplatelet therapy. Recent reviews highlight the Woven EndoBridge (WEB) as the most established platform, with newer devices such as Contour and Nautilus expanding this therapeutic class. Reported series demonstrate acceptable angiographic occlusion rates and a generally favorable safety profile, although retreatment and thromboembolic events remain relevant considerations [34, 35]. These devices could not be included in the present quantitative analysis due to limited ICA-terminus–specific data and heterogeneous outcome reporting; however, their growing role underscores the need for future location-focused comparative studies incorporating intrasaccular technologies.

## Limitations

The majority of included studies were retrospective in nature, introducing inherent risks of selection bias and inconsistent reporting of outcomes. The clinical grading and follow-up duration were inconsistently reported, and several studies lacked long-term outcome data, which hinders evaluation of durability and delayed complications. Additionally, due to variability in reporting and inclusion of case reports and small case series, some results may not be generalizable to broader patient populations.

## Conclusion

Satisfactory rates of complete occlusion and functional independence were achieved, particularly in unruptured cases. However, ruptured aneurysms were consistently associated with lower occlusion rates, higher mortality, and diminished clinical outcomes. These differences highlight rupture status as a central prognostic variable, and highlight the need for tailored treatment strategies. While endovascular therapy offers a viable and often effective approach, ongoing refinements in technique selection and device development remain essential to improve outcomes, especially in the acute rupture setting.

## Supplementary Information

Below is the link to the electronic supplementary material.

Supplementary Material 1

## Data Availability

No datasets were generated or analysed during the current study.
