# Supplementary material for: Endovascular management of internal carotid artery terminus aneurysms: a systematic review and meta-analysis
Source: Neurosurg Rev. 2026 Mar 14;49(1):276. doi: 10.1007/s10143-026-04210-3 (PMC12987796; doi:10.1007/s10143-026-04210-3)
Supplement: Supplementary file 1 — Supplementary Material 1 [file 10143_2026_4210_MOESM1_ESM.docx]

**Table S1:** Quality Assessment of Case Reports Using CARE Guidelines

| **Author(s)** | **Patient Information** | **Clinical Findings** | **Diagnostic Assessment** | **Therapeutic Interventions** | **Follow-up Outcomes** | **Discussion/Conclusions** | **Overall Quality** |
| --- | --- | --- | --- | --- | --- | --- | --- |
| **Benndorf et al. [11]** | Comprehensive | Detailed | Thorough | Well-documented | Reported | Needs Improvement | Moderate |
| **Zhenhai et al. [16]** | Comprehensive | Not Detailed | Thorough | Well-documented | Not Reported | Needs Improvement | Moderate |
| **Trivelato et al. [22]** | Comprehensive | Detailed | Thorough | Well-documented | Not Reported | Relevant | Moderate |
| **Mahajan et al. [24]** | Comprehensive | Detailed | Thorough | Well-documented | Reported | Relevant | High |
| **Spinelli et al. [27]** | Comprehensive | Detailed | Thorough | Well-documented | Reported | Relevant | High |

**Table S2:** ROBINS-I Assessment of the included Studies

| **Author(s)** | **Bias due to Confounding** | **Bias in Selection of Participants** | **Bias in Classification of Interventions** | **Bias due to Deviations from Intended Interventions** | **Bias due to Missing Data** | **Bias in Measurement of Outcomes** | **Bias in Selection of Reported Results** | **Overall Bias** |
| --- | --- | --- | --- | --- | --- | --- | --- | --- |
| Sakamoto et al. [12] | Low | Moderate | Low | Low | Moderate | Low | Low | Low |
| van Rooij et al. [13] | Moderate | Moderate | Low | Low | Low | Moderate | Low | Low |
| Uemura et al. [14] | Low | Moderate | Low | Low | Low | Low | Low | Low |
| Oishi et al. [15] | Low | Moderate | Low | Low | Low | Low | Low | Low |
| Zhou et al. [17] | Moderate | Moderate | Low | Low | Low | Low | Low | Low |
| Ban et al. [18] | Moderate | Moderate | Low | Low | Moderate | Moderate | Moderate | Moderate |
| Lee et al. [19] | Moderate | Low | Low | Low | Low | Low | Low | Low |
| Nossek et al. [20] | Moderate | Moderate | Low | Low | Moderate | Moderate | Moderate | Moderate |
| Morales-Valero et al. [21] | Moderate | Low | Low | Low | Low | Low | Low | Low |
| Pira et al. [23] | Serious | Moderate | Moderate | Moderate | Moderate | Moderate | Serious | Serious |
| Cagnazzo et al. [25] | Moderate | Low | Low | Low | Low | Low | Low | Low |
| Mahmoud et al. [26] | Moderate | Moderate | Low | Low | Low | Low | Moderate | Moderate |
